# Host‐Associated Differentiation of Gut Microbiota Among Four Crane Species Wintering in Rice Paddies Around Poyang Lake

**DOI:** 10.1002/ece3.74331

**Published:** 2026-09-23

**Authors:** Chaoyang Wang, Ke Mi, Ruiqing Shao, Xiaolan Luo, Deming Shen, Chao Zhang, Yiting Ai, Yingyuan Yu, Yankuo Li, Zhenyu Wang

**Affiliations:** ^1^ Key Laboratory of Biodiversity Conservation and Bioresources Utilization of Jiangxi Province, College of Life Sciences Jiangxi Normal University Nanchang Jiangxi China; ^2^ School of Life Sciences Central China Normal University Wuhan Hubei China; ^3^ Jiangxi Academy of Forestry Nanchang Jiangxi China; ^4^ Endoscopy Center The Affiliated Hospital of Jiangxi University of Chinese Medicine Nanchang Jiangxi China

**Keywords:** avian gut microbiota, core microbiota, host‐associated differentiation, phylosymbiosis, wintering cranes

## Abstract

Whether different bird species retain distinct gut microbial communities when using the same broad foraging habitat remains unclear. We used 16S rRNA gene amplicon sequencing to characterize fecal microbiota from 51 samples representing four crane species wintering in rice paddies around Poyang Lake, China. The sampled species were Siberian cranes (*Leucogeranus leucogeranus*), white‐naped cranes ( *Grus vipio* ), common cranes ( *G. grus* ), and hooded cranes ( *G. monacha* ). Taxonomic composition and all four alpha‐diversity indices differed among host species, while beta diversity showed partial host‐associated separation (PERMANOVA, *R* ^*2*^ = 0.19, *p* = 0.0033). Only eight of 124 core ASVs were shared across all four species, whereas 85 were species‐specific. Common and hooded cranes clustered together in both host and microbial trees, providing limited topological concordance. However, host phylogenetic distance was not significantly associated with microbial community dissimilarity overall (Mantel *r* = −0.293, *p* = 0.667). Predicted functional profiles retained a substantial shared component, with 77.8% of KEGG orthologs detected in all four species, although overall functional composition differed significantly among hosts. Potentially pathogen‐associated taxa occurred at generally low relative abundances and showed some variation among species. These findings show that shared use of rice paddies does not ensure microbial convergence and highlight the need to integrate dietary data when testing phylosymbiosis in wild birds.

## Graphical abstract

In this study, we used 16S rRNA gene amplicon sequencing to characterize the fecal microbiota of four crane species wintering in agricultural rice paddies at Poyang Lake, China: Siberian crane (*Leucogeranus leucogeranus*), hooded crane ( *Grus monacha* ), white‐naped crane ( *Grus vipio* ), and common crane ( *Grus grus* ). We found that gut microbial composition, alpha diversity, beta diversity, core microbiota, and indicator ASVs all differed among host species despite substantial overlap in winter foraging habitat. By contrast, predicted functional profiles were comparatively more conserved, with a large shared functional background across hosts. We also found no significant phylogenetic signal in microbial beta diversity, and potential pathogen‐associated taxa were generally present at low relative abundance but varied among species.

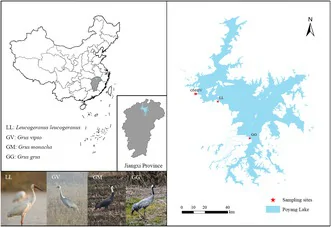

## 1 Introduction

Wild birds harbor diverse gut microbial communities that contribute to nutrient acquisition, energy metabolism, vitamin synthesis, immune regulation, and pathogen defense, thereby influencing host health and fitness (Hird et al. 2015; Sun et al. 2022). The diversity and composition of avian gut microbiota are shaped by both intrinsic and extrinsic factors. Intrinsic factors include host phylogeny, genetics, gut morphology, and immune function, whereas extrinsic factors include diet, habitat, climate, and behavior (Waite and Taylor 2014; Grond et al. 2018; Sun et al. 2022). Disentangling the relative contributions of these drivers remains a central challenge in microbial ecology. Previous studies have identified host‐associated patterns and, in some cases, phylogenetic signals in gut microbial communities (Groussin et al. 2020; Matheen et al. 2022; Pereira et al. 2024). Such correspondence between host phylogeny and microbial community relationships is termed phylosymbiosis and may arise partly through host filtering (Mazel et al. 2018; Groussin et al. 2020). At the same time, gut microbiota can respond rapidly to dietary and environmental variation, including seasonal change, climate, and ecological disturbance (Kohl 2012). Together, these findings raise an important question: when different bird species use a common broad habitat type, do their gut microbiota converge or remain distinct because of host‐associated filtering?

In the Poyang Lake region, hydrological regimes have shifted markedly in recent years, partly owing to reservoir regulation in the upper Yangtze River and climate variability (Li et al. 2020). Prolonged inundation during the growing season has reduced submerged macrophytes, thereby limiting natural food resources for wintering waterbirds (Zhang et al. 2022). As a result, many waterbirds, including cranes and geese, increasingly use agricultural habitats, particularly rice paddies, as alternative foraging grounds (Hou et al. 2020; Shen et al. 2025). Compared with natural wetlands, these habitats differ in food resources, environmental microbial communities, and human disturbance, all of which may influence avian gut microbiota (Li et al. 2019; Zhang et al. 2022). Because habitat use can influence avian fecal microbiota, rice paddies provide a common broad habitat type for comparing these species (Jarma et al. 2021; Lu et al. 2022; Sun et al. 2022). Thus, use of rice paddies does not imply identical diets, sampling‐site conditions, or microbial exposure. Host‐related factors may therefore continue to impose strong filtering effects within this common broad habitat type (Mazel et al. 2018; Maritan et al. 2024). This combination of a common broad habitat type and potential host‐related differences provides the ecological context for the present comparison.

The four crane species wintering at Poyang Lake provide a valuable comparative system for addressing these questions because they span several well‐established evolutionary lineages. Molecular phylogenies place the Siberian crane as sister to the remaining Gruinae (Krajewski et al. 2010). The white‐naped crane belongs to the Antigone species group, whereas the common and hooded cranes belong to the Americana species group. However, the four crane species can differ in diet composition, dietary breadth, and resource overlap within Poyang Lake (Hou et al. 2021). Under standardized captive conditions, all 15 crane species harbored species‐specific gut microbial communities, although only weak evidence of phylosymbiosis was detected (Trevelline et al. 2020). Field studies have characterized the fecal microbiota of hooded cranes and compared the gut microbial communities of white‐naped and red‐crowned cranes (Zhao et al. 2017; Gao et al. 2022). Other studies have documented microbial shifts associated with dietary change in Siberian cranes and with extreme drought in common cranes (Wang et al. 2023, 2024). However, field‐based comparisons that simultaneously consider multiple crane species, host phylogeny, and a common broad habitat type remain limited.

Here, we used 16S rRNA gene amplicon sequencing to compare fecal microbiota among four crane species wintering in rice paddies around Poyang Lake. Our central question was whether fecal microbial communities differed among host species and whether interspecific dissimilarity corresponded to host phylogenetic distance within this common broad habitat type. We predicted that host‐specific filtering would produce distinct taxonomic communities among species. A phylosymbiotic pattern would be supported if microbial dissimilarity increased with host phylogenetic distance, with the closely related common and hooded cranes showing greater similarity. Conversely, a strong homogenizing influence of rice‐paddy foraging would be expected to promote greater taxonomic and functional convergence. We therefore compared microbial diversity and composition, core ASVs, and predicted functional profiles, and explicitly tested the relationship between host phylogenetic and microbial dissimilarity. As a secondary conservation‐oriented assessment, we also screened for potential pathogen‐associated taxa.

## 2 Materials and Methods

### 2.1 Ethics Statement

All samples were collected noninvasively from freshly voided feces of free‐living cranes, and no animals were captured or handled.

### 2.2 Study Area and Sample Collection

**FIGURE 1:**
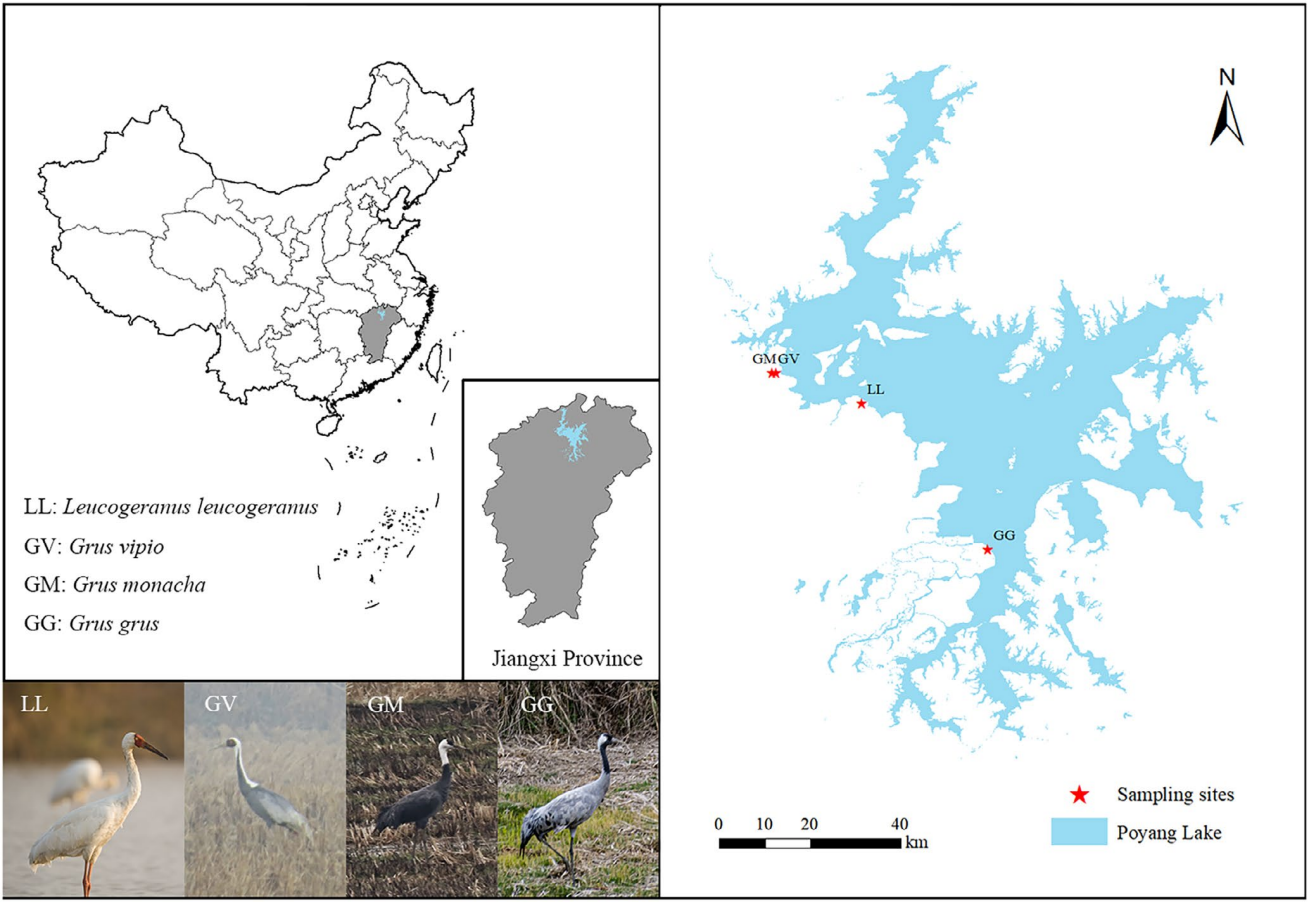
Study area and sampled crane species near Poyang Lake, China. Maps show the location of the study area in Jiangxi Province and sampling sites (red stars) around Poyang Lake (light blue). The GM and GV sites are geographically close but have distinct coordinates; both sites are identified on the map. Photographs depict the four sampled crane species: LL, Siberian cranes (*n* = 12); GV, white‐naped cranes (*n* = 15); GM, hooded cranes (*n* = 12); GG, common cranes (*n* = 12). Map source: Standard Map Service of China, approval number GS(2020)4619. Photo credits: Ruiqing Shao and Yankuo Li.

Poyang Lake, located in Jiangxi Province, China, is the largest freshwater lake in China and a key wintering site along the East Asian‐Australasian Flyway (Li et al. 2020; Wang, Gao, et al. 2021). During the sampling period, all four crane species included in this study overwintered in the Poyang Lake region and used rice paddies as foraging habitats. Fecal samples were collected from different rice paddies around Poyang Lake (Figure 1; Table S1). The GM and GV sampling sites were geographically close but had distinct coordinates. All sampling fields represented the same broad habitat type, namely rice paddies, and no marked differences in broad‐scale habitat characteristics were apparent during field observations. Sampling dates, geographic coordinates, and sample numbers for each sampling field are provided in Table S1.

We collected 51 fresh fecal samples from four crane species foraging in rice paddies: *Leucogeranus leucogeranus* (LL, *n* = 12), *Grus vipio* (GV, *n* = 15), *G. grus* (GG, *n* = 12), and *G. monacha* (GM, *n* = 12) (Figure 1). Sampling was conducted from November to December 2020. Before sample collection, experienced observers used spotting scopes to identify crane species and continuously monitored the focal flocks. Although mixed‐species flocks occurred in the study area, samples were collected only when the focal flock consisted of a single crane species. After an identified bird was observed defecating and the flock had moved away, observers immediately entered the field and collected the freshly voided feces from the observed location. Species identity was therefore assigned primarily through direct observation of defecation, with fecal morphology used only as Supporting Information based on the field team's previous experience. To reduce the risk of collecting multiple samples from the same individual, sampling points were separated by at least 5 m (Zhang et al. 2012). Material was collected from the interior of each dropping to minimize environmental contamination, immediately frozen on dry ice, and stored at −80°C until DNA extraction.

### 2.3 DNA Extraction and Sequencing

Bacterial DNA was extracted from fecal samples using the QIAamp DNA Stool Mini Kit (QIAGEN, Germany) following the manufacturer's protocol. The V3–V4 region of the 16S rRNA gene was amplified using barcoded primers 343F (5′‐TACGGRAGGCAGCAG‐3′) and 798R (5′‐AGGGTATCTAATCCT‐3′). PCR reactions (25 μL) contained 8.5 μL nuclease‐free water, 12.5 μL 2× TaKaRa Taq polymerase, 1 μL of each primer, and 2 μL DNA template. Thermal cycling conditions were: initial denaturation at 95°C for 5 min; 30 cycles of 95°C for 30 s, 55°C for 60 s, and 72°C for 60 s; followed by a final extension at 72°C for 5 min. Amplicons were checked on 1.5% agarose gels (100 V for 60 min), purified using the Agencourt AMPure XP kit (Beckman Coulter), and sequenced on an Illumina platform using a 2 × 300‐bp paired‐end configuration.

### 2.4 Bioinformatics Analysis

Raw paired‐end reads were processed using the DADA2 pipeline (Callahan et al. 2016). After quality filtering and truncation, paired reads were merged, and chimeric sequences were removed to obtain high‐resolution amplicon sequence variants (ASVs). Taxonomic assignment was performed with a naive Bayes classifier trained on the SILVA v138 reference database. A phylogenetic tree was constructed using FastTree based on multiple sequence alignments generated by DECIPHER (Wright 2016). The ASV abundance table, taxonomic annotations, phylogenetic tree, and sample metadata were integrated into a *phyloseq* object (McMurdie and Holmes 2013). To minimize the influence of sequencing noise and spurious features, ASVs present in only one sample or with fewer than 100 reads were removed, and ASVs that could not be classified at the phylum level were excluded. Downstream analyses were based on this filtered dataset.

### 2.5 Statistical Analysis

Before alpha‐diversity analysis, each sample was rarefied to 41,298 sequences using the rarefy_even_depth function in phyloseq (McMurdie and Holmes 2013). Observed richness, ACE, Shannon, and Simpson indices were then calculated at the ASV level. Differences in alpha diversity among host species were assessed using Kruskal–Wallis tests, followed by Dunn's post hoc comparisons with false discovery rate (FDR) correction. Differences in the relative abundances of major bacterial phyla and genera among host species were also evaluated using Kruskal–Wallis tests, followed, where appropriate, by Dunn's post hoc comparisons with FDR correction. Beta diversity was evaluated using Bray–Curtis dissimilarity and visualized by principal coordinates analysis (PCoA). The effect of host species on microbial community structure was assessed using PERMANOVA (Anderson 2001) with 9999 permutations. Homogeneity of multivariate dispersion among host species was assessed using PERMDISP (the betadisper function in vegan, based on distances to group spatial medians) with 9999 permutations.

To evaluate host–microbiota phylogenetic concordance, partial mitochondrial cytochrome c oxidase subunit I (COI) sequences were downloaded from NCBI GenBank for *Grus vipio* (KF939579.1), *G. grus* (GU571917.1), *Leucogeranus leucogeranus* (KJ862169.1), and *G. monacha* (KJ862165.1). The four sequences were assembled into a nucleotide alignment in MEGA11 (Tamura et al. 2021), and a neighbor‐joining (NJ) tree was reconstructed using the Maximum Composite Likelihood model, assuming uniform rates among sites and applying pairwise deletion to gaps and missing data. Node support was assessed using 1000 bootstrap replicates. The resulting host tree was imported into R, and pairwise host phylogenetic distances were calculated using the cophenetic.phylo function in the ape package. To obtain species‐level microbial distances, ASV counts were pooled by host species, converted to relative abundances, and used to calculate Bray–Curtis dissimilarities in phyloseq. The association between host phylogenetic distances and microbial community dissimilarities was evaluated using a Pearson Mantel test in vegan with 9999 permutations. UPGMA and NJ microbial dendrograms were constructed from the species‐level Bray–Curtis distance matrix using average‐linkage hierarchical clustering and the nj function in ape, respectively. Cophylogenetic relationships between the host and microbial trees were visualized using the cophylo function in phytools.

Taxonomic biomarkers associated with each host species were identified using LEfSe, with an LDA score threshold > 3 (Segata et al. 2011). Functional profiles were inferred using PICRUSt2 and annotated to KEGG orthologs (Douglas et al. 2020). Differences in predicted functional composition among host species were assessed using Bray–Curtis dissimilarities, PCoA, and PERMANOVA, followed, where appropriate, by pairwise PERMANOVA with Benjamini–Hochberg FDR correction. Because these functions were predicted from 16S rRNA gene data rather than directly measured, they were interpreted as potential rather than realized metabolic functions. Core microbiota were defined within each host species using a prevalence–abundance criterion: ASVs had to occur in at least 50% of samples and have a mean relative abundance of at least 0.1%. Because no universal threshold defines a core microbiome, these cutoffs were selected as an operational balance between within‐species consistency and the exclusion of very rare taxa, and were stated explicitly to ensure reproducibility (Neu et al. 2021). Potentially pathogen‐associated taxa were screened based on taxonomic annotations and classifications reported in previous studies; these taxa and their corresponding literature sources are listed in Table S2. Their occurrence and relative abundances were summarized descriptively for each host species. Statistical significance was set at *p* < 0.05; FDR‐adjusted *p* values were used for post hoc comparisons. Unless otherwise stated, all analyses were conducted in R v4.4.3.

## 3 Results

After quality control and before rarefaction, the 51 fecal samples yielded an average of 56,653 high‐quality sequences per sample. DADA2 denoising identified 23,660 ASVs. After excluding ASVs present in only one sample, with < 100 reads, or lacking phylum‐level annotation, 2312 ASVs remained and were used for downstream analyses. Among these, 1853 ASVs were annotated at the genus level and 1269 at the species level.

### 3.1 Gut Microbial Community Composition, Core Microbiota, and Indicator ASVs Differ Among Crane Species

**FIGURE 2:**
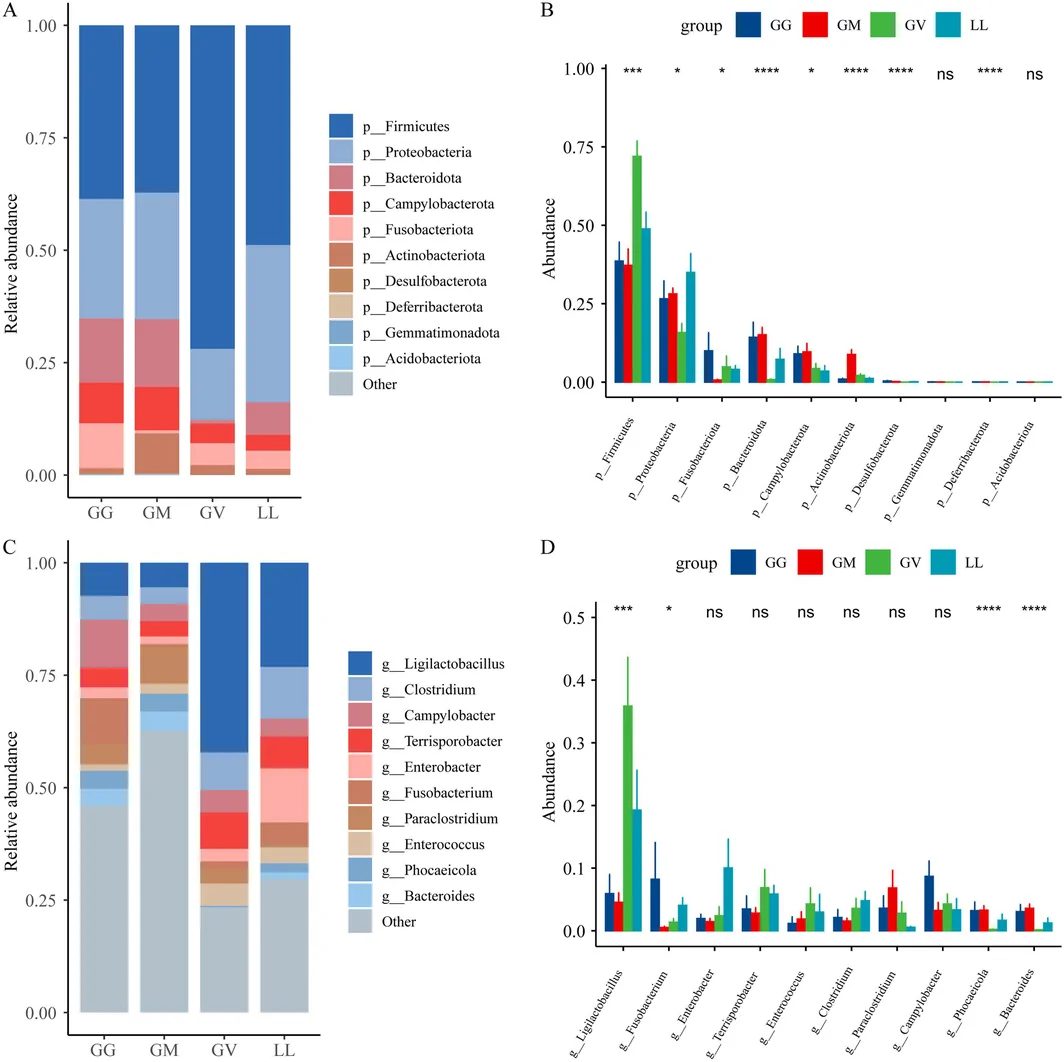
(A) Relative abundance of major bacterial phyla in common cranes (GG), hooded cranes (GM), white‐naped cranes (GV), and Siberian cranes (LL). (B) Relative abundance of selected phyla compared among species. (C) Relative abundance of major bacterial genera in the four crane species. (D) Relative abundance of selected genera compared among species. Differences among host species were evaluated using Kruskal–Wallis tests followed by Dunn's post hoc comparisons with false discovery rate (FDR) correction. ns, FDR‐adjusted *p* ≥ 0.05; *, FDR‐adjusted *p* < 0.05; **, FDR‐adjusted *p* < 0.01; ***, FDR‐adjusted *p* < 0.001; and ****, FDR‐adjusted *p* < 0.0001.

Across the four crane species, gut bacterial communities were dominated by Firmicutes (49.15% ± 16.06%) and Proteobacteria (26.36% ± 7.93%), followed by Bacteroidota (9.37% ±6.69%), Campylobacterota (6.62% ± 3.16%), Fusobacteriota (4.90% ± 3.84%), and Actinobacteriota (3.28% ± 3.72%; Figure 2A). The relative abundances of these six dominant phyla differed significantly among host species, although the strength of statistical support varied among phyla (Kruskal–Wallis tests, all FDR‐adjusted *p* < 0.05; Figure 2B). Genus‐level composition also varied among species (Figure 2C,D). White‐naped cranes had a high relative abundance of *Ligilactobacillus* (35.85%), whereas Siberian cranes had a comparatively high relative abundance of *Clostridium sensu stricto 1* (9.56%). Common cranes had relatively high abundances of *Campylobacter* (8.68%) and *Fusobacterium* (8.22%), while hooded cranes had relatively high abundances of *Paraclostridium* (6.83%) and *Helicobacter* (4.07%). Among the 10 dominant genera shown in Figure 2D, Ligilactobacillus, Fusobacterium, Phocaeicola, and Bacteroides differed significantly among host species (FDR‐adjusted *p* < 0.05), whereas differences in the remaining six genera were not significant. LEfSe further identified distinct taxonomic biomarkers associated with each crane species (Figure S1).

**FIGURE 3:**
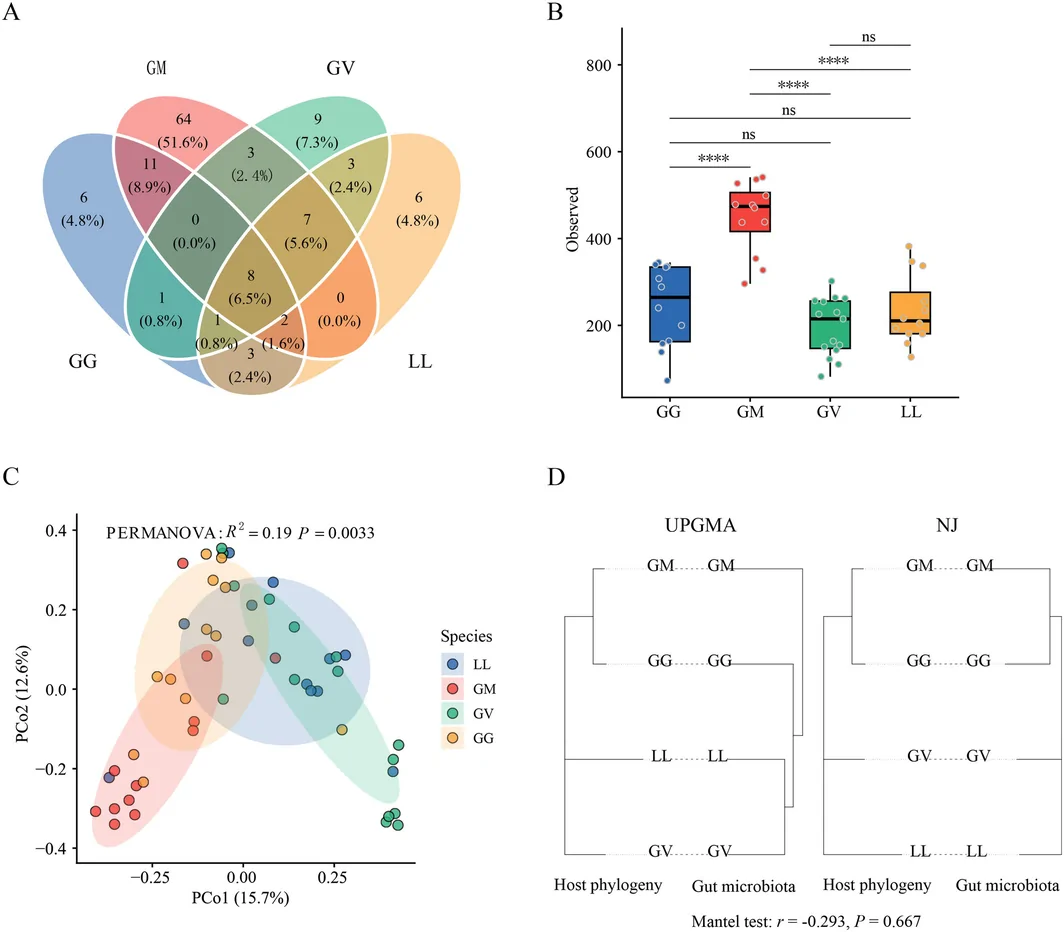
(A) Venn diagram of shared core amplicon sequence variants (ASVs) among common cranes (GG), hooded cranes (GM), white‐naped cranes (GV), and Siberian cranes (LL). (B) Observed ASV richness in the four crane species. Boxes show the median and interquartile range, whiskers extend to 1.5 times the interquartile range, and points represent individual samples. Overall differences were evaluated using Kruskal–Wallis tests, followed by pairwise Dunn's post hoc comparisons with false discovery rate (FDR) correction. (C) Principal coordinates analysis (PCoA) based on Bray–Curtis dissimilarities showing gut microbiota differences among the four crane species. Permutational multivariate analysis of variance (PERMANOVA) showed a significant host‐species effect (*R* ^*2*^ = 0.19, *p* = 0.0033). A permutational test for homogeneity of multivariate dispersions (PERMDISP) detected no significant difference in dispersion among host species (*F* = 0.989, *p* = 0.409). (D) Cophylogenetic comparisons between the host phylogenetic tree and gut microbial dendrograms constructed from species‐level Bray–Curtis dissimilarities using the unweighted pair‐group method with arithmetic mean (UPGMA; left panel) and neighbor joining (NJ; right panel). Lines connect corresponding crane species between the host and microbial trees. The Mantel test showed no significant association between host phylogenetic distance and microbial community dissimilarity (*r* = −0.293, *p* = 0.667). ns, FDR‐adjusted *p* ≥ 0.05; *, FDR‐adjusted *p* < 0.05; **, FDR‐adjusted *p* < 0.01; ***, FDR‐adjusted *p* < 0.001; and ****, FDR‐adjusted *p* < 0.0001.

Core microbiota analysis showed limited overlap among host species. Using a prevalence threshold of ≥ 50% and a mean relative abundance threshold of ≥ 0.1%, the union of the four host‐specific core sets contained 124 ASVs. Of these, only 8 (6.5%) were shared by all four species, whereas 85 (68.5%) were species‐specific and 31 (25.0%) were shared by two or three species (Figure 3A). The number of species‐specific core ASVs differed markedly among hosts. Hooded cranes harbored 64 such ASVs, compared with 6 in Siberian cranes, 6 in common cranes, and 9 in white‐naped cranes. Indicator analysis identified 606 significant indicator ASVs (*p* < 0.05), of which 396 (65.3%) were associated with a single host species. Hooded cranes had the largest number of species‐specific indicators (304 ASVs), compared with Siberian cranes (26), common cranes (38), and white‐naped cranes (28). At least 80 indicator ASVs overlapped with the core ASV set, including 77 species‐specific core ASVs.

### 3.2 Gut Microbial Diversity Differs Among Host Species, With Limited Phylogenetic Concordance

Alpha diversity differed significantly among the four crane species (Figures 3B and S2). Overall Kruskal–Wallis tests detected significant differences among host species in observed richness, ACE, Shannon, and Simpson indices (all *p* < 0.01). Subsequent Dunn's tests with FDR correction showed that hooded cranes had significantly higher values than the other three species across all four indices (*p* < 0.05). Most pairwise differences among Siberian cranes, common cranes, and white‐naped cranes were not significant. Significant differences between Siberian and white‐naped cranes were detected only for the Shannon and Simpson indices (FDR‐adjusted *p* < 0.05).

Beta diversity analysis further revealed host‐associated differentiation in gut microbial community structure. PCoA based on Bray–Curtis dissimilarities showed partial separation of samples by host species (Figure 3C), and PERMANOVA confirmed significant differences in community composition among species (*R* ^*2*^ = 0.19, *p* = 0.0033). PERMDISP did not detect significant heterogeneity in multivariate dispersion among host species for Bray–Curtis dissimilarities (*F* = 0.989, *p* = 0.409). Notably, common and hooded cranes (GG and GM) formed a sister grouping in the host phylogeny and clustered together in both the UPGMA‐ and NJ‐based microbial dendrograms (Figure 3D), indicating partial topological concordance between host and microbial relationships. However, the Mantel test detected no significant overall association between host phylogenetic distance and microbial community dissimilarity (*r* = −0.293, *p* = 0.667). Thus, the clustering of GG and GM provided limited descriptive topological concordance, but this four‐species comparison did not provide statistical support for an overall phylosymbiotic pattern.

### 3.3 Host‐Associated Differences in Predicted Gut Microbial Functions

**FIGURE 4:**
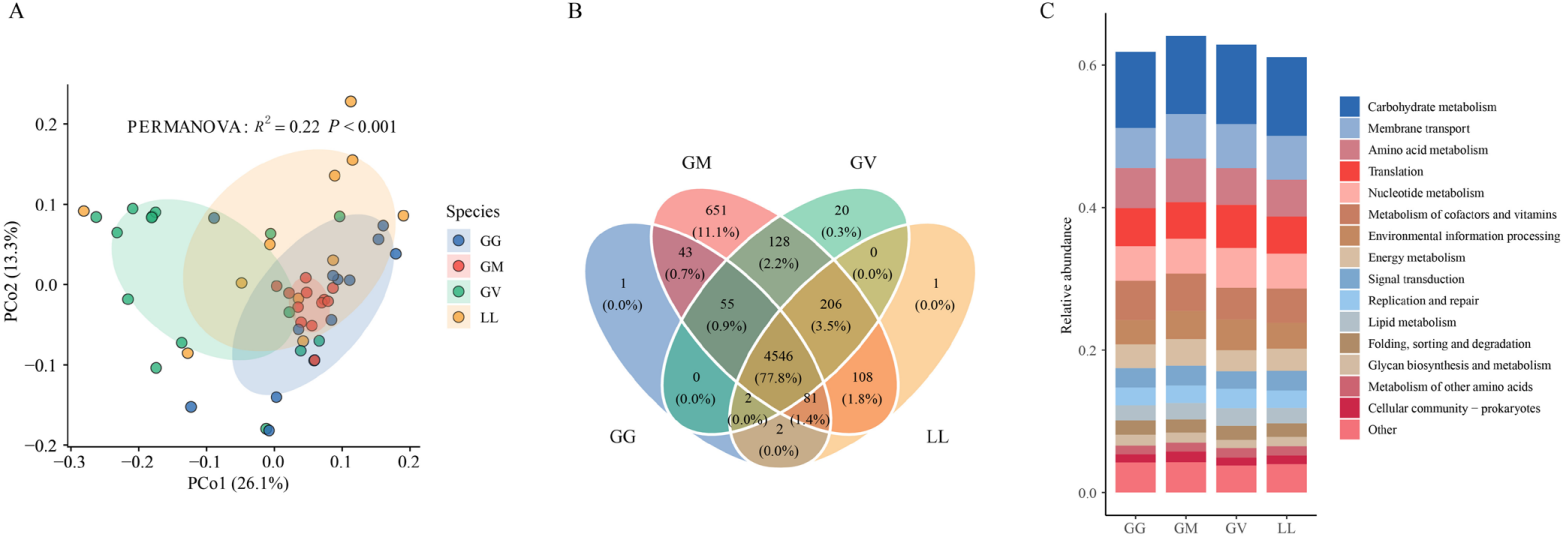
(A) PCoA of predicted KO profiles based on Bray–Curtis distances showing functional differences among common cranes (GG), hooded cranes (GM), white‐naped cranes (GV), and Siberian cranes (LL). PERMANOVA showed significant differences in predicted functional composition among host species (*R* ^*2*^ = 0.22, *p* < 0.001). (B) Venn diagram of shared predicted KOs among the four crane species. (C) Relative abundance of major KEGG level 2 functional categories predicted by PICRUSt2.

PICRUSt2‐based inference revealed interspecific differences in predicted gut microbial functional profiles. PCoA of KEGG ortholog (KO) profiles showed partial separation by host species (Figure 4A), and PERMANOVA confirmed significant differences in predicted functional composition among species (*R* ^*2*^ = 0.22, *p* < 0.001). Pairwise PERMANOVA with FDR correction detected significant differences in five of the six species pairs; only Siberian and common cranes did not differ significantly (Table 1). Despite these differences, 4546 of 5844 predicted KOs (77.8%) were shared across all four species. Hooded cranes had the largest set of species‐specific predicted KOs (651 KOs; Figure 4B).

**TABLE 1:** Pairwise PERMANOVA results for predicted gut microbial functional profiles (KO level) among the four crane species.

Comparison	*F*	*R* ^*2*^	FDR‐adjusted *p* value
LL vs. GM	5.822	0.209	**< 0.001**
LL vs. GV	3.292	0.116	**0.018**
LL vs. GG	1.510	0.064	0.160
GM vs. GV	7.553	0.232	**< 0.001**
GM vs. GG	4.259	0.162	**< 0.001**
GV vs. GG	5.162	0.171	**0.002**

*Note:* Significant adjusted *p* values are shown in bold. LL, Siberian crane; GM, hooded crane; GV, white‐naped crane; GG, common crane. *p* values were adjusted across the six pairwise comparisons using the Benjamini–Hochberg FDR procedure.

At KEGG Level 2, predicted functional profiles were broadly similar among hosts and were dominated by carbohydrate metabolism, membrane transport, and amino acid metabolism (Figure 4C). Other relatively abundant categories included translation, nucleotide metabolism, metabolism of cofactors and vitamins, environmental information processing, and energy metabolism. Overall, the predicted functional profiles contained a substantial shared component, although their composition differed significantly among host species.

### 3.4 Occurrence and Relative Abundance of Taxa Annotated as Potentially Pathogen‐Associated

**FIGURE 5:**
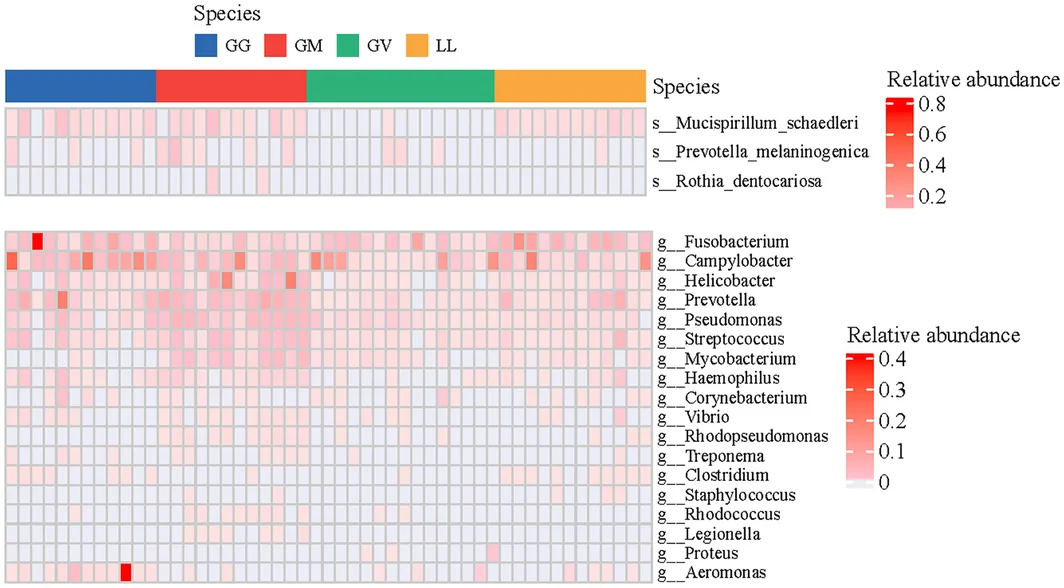
Heatmaps showing the relative abundance of representative bacterial taxa among common cranes (GG), hooded cranes (GM), white‐naped cranes (GV), and Siberian cranes (LL). The upper panel shows species‐level taxa, and the lower panel shows genus‐level taxa. Columns represent individual samples, rows represent taxa, and colors indicate relative abundance.

Taxa annotated as potentially pathogen‐associated occurred at generally low relative abundances across the four crane species. We detected 18 such genera and three such species (Table S2). The number of genera per host ranged from 10 in Siberian cranes to 13 in hooded cranes, whereas white‐naped and common cranes each harbored 11 genera. Four genera, *Clostridium*, *Prevotella*, *Streptococcus*, and *Mycobacterium*, occurred in more than half of the samples. Among these, *Clostridium* had the highest mean relative abundance and exceeded 2% in all four hosts (LL: 9.48%; GM: 2.70%; GV: 6.65%; GG: 3.49%). At the species level, *Mucispirillum schaedleri* and *Prevotella melaninogenica* were detected in all four crane species, whereas *Rothia dentocariosa* was detected only in hooded cranes. The combined relative abundance of the annotated genera was highest in common cranes (GG: 12.55%), followed by Siberian cranes (LL: 11.24%), hooded cranes (GM: 7.81%), and white‐naped cranes (GV: 7.21%; Figure 5).

## 4 Discussion

This study presents a comparative analysis of gut microbiota in four crane species wintering in agricultural habitats around Poyang Lake. All four species were sampled from rice paddies during the same wintering period, providing a common broad habitat context for comparison. However, this similarity in macrohabitat type does not imply identical local environmental exposure. Gut microbial composition, alpha and beta diversity, core microbiota, indicator ASVs, and predicted functional profiles differed among the sampled host populations. These findings demonstrate species‐associated microbial differentiation among cranes using the same broad habitat type. Because each host species was sampled at a different site, host‐species effects cannot be separated from local site effects.

### 4.1 Community Composition and Core Microbiota Structure

Across all four crane species, the gut microbiota was dominated by Firmicutes and Proteobacteria, with additional contributions from Bacteroidota, Campylobacterota, Fusobacteriota, and Actinobacteriota. This overall taxonomic pattern is consistent with previous studies of cranes and other waterbirds (Wang, Huang, et al. 2021; Fu et al. 2020; Zhao et al. 2021; Wang et al. 2018). Members of Firmicutes and Bacteroidota are commonly associated with the degradation of complex carbohydrates and other plant‐derived substrates, consistent with the predominantly herbivorous winter diet of cranes in the Poyang Lake region (Thomas et al. 2011; Lu et al. 2022). Proteobacteria are likewise common components of avian gut microbiota, although their ecological roles can vary substantially among taxa and environmental contexts (Grond et al. 2018; Sun et al. 2022). Although dominant phyla were broadly shared among host species (Zou et al. 2025), their relative abundances differed significantly. This pattern indicates taxonomic differentiation among crane populations using rice paddies, although host species and local site effects cannot be separated in the present design.

Core microbiota analysis provided additional evidence of species‐associated differentiation. Only a small proportion of core ASVs were shared across all four crane species, whereas the majority were species‐specific. Core taxa are often interpreted as recurrent members of the gut microbiota (Neu et al. 2021), but temporal persistence cannot be inferred from cross‐sectional sampling. The limited overlap observed here is therefore consistent with host‐associated filtering, although unmeasured dietary and site variation may also have contributed (Hird et al. 2015; Bodawatta et al. 2021; Pereira et al. 2024). The overlap between indicator ASVs and host‐specific core ASVs further shows that some taxa associated with interspecific differentiation were also frequently detected within the corresponding host population. Together, these results indicate consistent species‐associated patterns at the time of sampling, rather than long‐term stability or persistent ecological relationships (Kohl et al. 2018).

### 4.2 Diversity Patterns, Host‐Associated Effects, and Phylogenetic Signal

We detected clear interspecific differences in alpha diversity, with hooded cranes exhibiting the highest microbial richness and evenness among the four species. This pattern is notable because hooded cranes also harbored the largest numbers of species‐specific core ASVs and indicator taxa, suggesting that their gut microbiota was both more diverse and more distinctive than that of the other crane species. Variation in alpha diversity among sympatric bird species is often linked to differences in diet, habitat use, and environmental exposure, even when broad habitat types overlap (Matheen et al. 2022; Pereira et al. 2024). These differences among crane populations using the same broad habitat type are consistent with host‐related influences. However, unmeasured variation among sampling sites may also have contributed to the observed alpha‐diversity patterns (Costantini et al. 2024).

Beta‐diversity analyses further supported host‐associated differentiation. Microbial communities showed partial clustering by host species, and PERMANOVA confirmed significant interspecific differences in community composition. Common and hooded cranes clustered together in both the host phylogeny and the UPGMA‐ and NJ‐based microbial dendrograms, providing limited descriptive concordance. However, the Mantel test did not detect an overall association between host phylogenetic distance and microbial community dissimilarity. The GG‐GM correspondence alone therefore does not provide statistical support for phylosymbiosis across the four species. This conclusion remains constrained by the small number of host species and limited phylogenetic breadth, which reduce the power of comparative tests (Song et al. 2020; Trevelline et al. 2020).

Shared use of rice paddies should not be equated with identical food intake. Previous DNA metabarcoding at Poyang Lake demonstrated marked interspecific and interannual dietary variation among the same four crane species (Hou et al. 2021). Siberian cranes shifted between *Vallisneria* tubers and agricultural resources, including rice seeds and lotus rhizomes. White‐naped and hooded cranes relied more strongly on grassland plants and showed the greatest dietary overlap, whereas common cranes displayed a more generalist foraging strategy. These ecological differences are relevant because diet can strongly shape gut microbiota composition and assembly in Poyang Lake waterbirds (Wang et al. 2022). More broadly, diet and land use can modify phylogenetic or host‐associated microbial patterns in wild birds (Loo et al. 2019; San Juan et al. 2020). Diet shifts have also been associated with microbial changes in Siberian cranes (Wang et al. 2023). Because food intake was not directly characterized during our sampling period, the observed microbial differences cannot be attributed exclusively to host‐intrinsic filtering. Unmeasured dietary variation may also weaken or obscure correspondence between host phylogeny and microbial community structure. Our findings therefore support host‐associated differentiation within a shared broad habitat type, but they do not isolate the effects of host phylogeny from diet.

### 4.3 Functional Prediction and Host‐Associated Metabolic Signatures

Predicted functional profiles showed a contrasting pattern. Despite clear taxonomic differentiation, most predicted functions were shared across host species. Major KEGG categories were dominated by carbohydrate metabolism, membrane transport, and amino acid metabolism, indicating a broadly conserved predicted functional background (Gao et al. 2023). This taxonomic‐functional decoupling is consistent with functional redundancy among gut microbial communities. Such redundancy may allow taxonomically distinct communities to support overlapping metabolic functions across host species (Trevelline et al. 2020; Kubovčiak and Kreisinger 2025). The reported use of plant‐derived foods by these cranes may impose partially overlapping functional demands on their gut microbiota (Lu et al. 2022; Wang et al. 2022). At the same time, we detected significant differences in overall functional composition among host species, indicating that taxonomic turnover is not entirely functionally neutral. These differences may reflect subtle variation in diet, physiology, or microbial interactions among host species (Trevelline et al. 2020; Gao et al. 2021; Wang et al. 2023). Importantly, because these functional profiles were inferred from 16S rRNA gene data using PICRUSt2, they represent predicted metabolic potential rather than directly measured activity. Therefore, these results should be interpreted cautiously, and future studies using metagenomic or metatranscriptomic approaches will be necessary to validate these functional patterns.

### 4.4 Taxa Annotated as Potentially Pathogen‐Associated and Implications for Monitoring

Taxa annotated as potentially pathogen‐associated were detected in all four crane species, but most occurred at low relative abundances. Common cranes had the highest combined relative abundance of the annotated genera. However, because these values were summarized descriptively, they should not be treated as evidence of statistically supported interspecific differences in disease exposure or susceptibility. Moreover, genus‐level taxonomic assignments cannot distinguish pathogenic from non‐pathogenic strains, and detection in feces does not demonstrate infection or disease (Benskin et al. 2009). Accordingly, our data provide a descriptive baseline for future surveillance rather than a direct assessment of disease risk. Future studies combining strain‐level identification with host health indicators and environmental data will be needed to evaluate the conservation relevance of these taxa.

## 5 Conclusions

Our study identified species‐associated differences in gut microbial composition, diversity, core membership, indicator ASVs, and predicted functional profiles among four crane species wintering around Poyang Lake. Although all four species used rice paddies as a broad foraging habitat, their gut microbiota did not converge completely. However, the four host species were sampled at four distinct sites, with one site per species, so host‐species differences cannot be separated from local environmental variation. The study is further limited by the taxonomic resolution of 16S rRNA gene sequencing, the use of predicted rather than measured functions, the absence of direct dietary data, and the small number of host species. Future studies should combine replicated multispecies sampling at the same sites with diet metabarcoding, local environmental measurements, metagenomics, and longitudinal sampling. Such designs will better resolve the relative contributions of host identity, diet, local environment, and temporal variation to avian gut microbiota.

## Author Contributions

**Chaoyang Wang:** conceptualization (equal), data curation (equal), formal analysis (equal), investigation (equal), methodology (equal), visualization (equal), writing – original draft (equal), writing – review and editing (equal). **Ke Mi:** conceptualization (equal), data curation (equal), formal analysis (equal), methodology (equal), writing – review and editing (equal). **Ruiqing Shao:** data curation (equal), investigation (equal), visualization (equal), writing – review and editing (equal). **Xiaolan Luo:** data curation (equal), investigation (equal). **Deming Shen:** data curation (equal), investigation (equal). **Chao Zhang:** formal analysis (equal), visualization (equal), writing – review and editing (equal). **Yiting Ai:** data curation (equal), investigation (equal). **Yingyuan Yu:** data curation (equal). **Yankuo Li:** project administration (equal), supervision (equal), writing – review and editing (equal). **Zhenyu Wang:** project administration (equal), supervision (equal), writing – review and editing (equal).

## Funding

This study was supported by the National Natural Science Foundation of China (No. 32160244, 32260275) and the Jiangxi Provincial Key Research and Development Program (grant No. 20261BCF320019).

## Conflicts of Interest

The authors declare no conflicts of interest.

## Supporting information


**Figure S1:** LEfSe‐identified taxonomic biomarkers distinguishing four crane species. Histogram of linear discriminant analysis (LDA) scores (log10) for taxa significantly enriched in each host (GG, GM, GV, LL), showing species‐specific microbial biomarkers detected by LEfSe.
**Figure S2:** Alpha diversity of gut microbiota across four crane species. (A–C) Boxplots of the abundance‐based coverage estimator (ACE), Shannon index, and Simpson index, respectively. Overall differences among host species were evaluated using Kruskal–Wallis tests, followed by pairwise Dunn's post hoc comparisons with false discovery rate (FDR) correction. ns, FDR‐adjusted *p* ≥ 0.05; *, FDR‐adjusted *p* < 0.05; **, FDR‐adjusted *p* < 0.01; ***, FDR‐adjusted *p* < 0.001; and ****, FDR‐adjusted *p* < 0.0001.
**Table S1:** Sampling information for fecal samples collected from four wintering crane species around Poyang Lake, China.
**Table S2:** Taxa annotated as potentially pathogen‐associated. List of 15 potential pathogen‐associated genera and three species detected across four crane species (LL, GM, GV, GG), with their relative abundances and associated references.

## Data Availability

The raw 16S rRNA gene amplicon sequencing data generated in this study are available in the NCBI Sequence Read Archive under BioProject accession PRJNA1448014.
