# Supplementary material for: Host‐Associated Differentiation of Gut Microbiota Among Four Crane Species Wintering in Rice Paddies Around Poyang Lake
Source: Ecol Evol. 2026 Sep 23;16(9):e74331. doi: 10.1002/ece3.74331 (PMC13598929; doi:10.1002/ece3.74331)
Supplement: Supplementary file 1 [file ECE3-16-e74331-s001.docx]

**Supplementary figure and table legends**

**Figure S1** LEfSe-identified taxonomic biomarkers distinguishing four crane species. Histogram of linear discriminant analysis (LDA) scores (log10) for taxa significantly enriched in each host (GG, GM, GV, LL), showing species-specific microbial biomarkers detected by LEfSe.

**Figure S2** Alpha diversity of gut microbiota across four crane species. (A–C) Boxplots of the abundance-based coverage estimator (ACE), Shannon index, and Simpson index, respectively. Overall differences among host species were evaluated using Kruskal–Wallis tests, followed by pairwise Dunn’s post hoc comparisons with false discovery rate (FDR) correction. ns, FDR-adjusted *P* ≥ 0.05; *, FDR-adjusted *P* < 0.05; **, FDR-adjusted *P* < 0.01; ***, FDR-adjusted *P* < 0.001; and ****, FDR-adjusted *P* < 0.0001.

**Table S1** Sampling information for fecal samples collected from four wintering crane species around Poyang Lake, China.

**Table S2** Taxa annotated as potentially pathogen-associated. List of 15 potential pathogen-associated genera and three species detected across four crane species (LL, GM, GV, GG), with their relative abundances and associated references.

**Figure S1**

**Figure S2**

**Table S1 Sampling information for fecal samples collected from four wintering crane species around Poyang Lake, China.**

| **Sample ID** | **Species** | **Group** | **Sampling date** | **Latitude** | **Longitude** | **Habitat type** |
| --- | --- | --- | --- | --- | --- | --- |
| BH01 | *Leucogeranus leucogeranus* | LL | 2020-12-06 | 29.071566 N | 116.053165 E | rice paddy |
| BH02 | *Leucogeranus leucogeranus* | LL | 2020-12-06 | 29.071566 N | 116.053165 E | rice paddy |
| BH03 | *Leucogeranus leucogeranus* | LL | 2020-12-06 | 29.071566 N | 116.053165 E | rice paddy |
| BH04 | *Leucogeranus leucogeranus* | LL | 2020-12-06 | 29.071566 N | 116.053165 E | rice paddy |
| BH05 | *Leucogeranus leucogeranus* | LL | 2020-12-06 | 29.071566 N | 116.053165 E | rice paddy |
| BH06 | *Leucogeranus leucogeranus* | LL | 2020-12-06 | 29.071566 N | 116.053165 E | rice paddy |
| BH07 | *Leucogeranus leucogeranus* | LL | 2020-12-06 | 29.071566 N | 116.053165 E | rice paddy |
| BH08 | *Leucogeranus leucogeranus* | LL | 2020-12-06 | 29.071566 N | 116.053165 E | rice paddy |
| BH09 | *Leucogeranus leucogeranus* | LL | 2020-12-06 | 29.071566 N | 116.053165 E | rice paddy |
| BH10 | *Leucogeranus leucogeranus* | LL | 2020-12-06 | 29.071566 N | 116.053165 E | rice paddy |
| BH11 | *Leucogeranus leucogeranus* | LL | 2020-12-06 | 29.071566 N | 116.053165 E | rice paddy |
| BH12 | *Leucogeranus leucogeranus* | LL | 2020-12-06 | 29.071566 N | 116.053165 E | rice paddy |
| BTH01 | *Grus monacha* | GM | 2020-12-13 | 29.133538 N | 115.8831759 E | rice paddy |
| BTH02 | *Grus monacha* | GM | 2020-12-13 | 29.133538 N | 115.8831759 E | rice paddy |
| BTH03 | *Grus monacha* | GM | 2020-12-13 | 29.133538 N | 115.8831759 E | rice paddy |
| BTH04 | *Grus monacha* | GM | 2020-12-13 | 29.133538 N | 115.8831759 E | rice paddy |
| BTH05 | *Grus monacha* | GM | 2020-12-13 | 29.133538 N | 115.8831759 E | rice paddy |
| BTH06 | *Grus monacha* | GM | 2020-12-13 | 29.133538 N | 115.8831759 E | rice paddy |
| BTH07 | *Grus monacha* | GM | 2020-12-13 | 29.133538 N | 115.8831759 E | rice paddy |
| BTH08 | *Grus monacha* | GM | 2020-12-13 | 29.133538 N | 115.8831759 E | rice paddy |
| BTH09 | *Grus monacha* | GM | 2020-12-13 | 29.133538 N | 115.8831759 E | rice paddy |
| BTH10 | *Grus monacha* | GM | 2020-12-13 | 29.133538 N | 115.8831759 E | rice paddy |
| BTH11 | *Grus monacha* | GM | 2020-12-13 | 29.133538 N | 115.8831759 E | rice paddy |
| BTH12 | *Grus monacha* | GM | 2020-12-13 | 29.133538 N | 115.8831759 E | rice paddy |
| BZ01 | *Grus vipio* | GV | 2020-12-28 | 29.133513 N | 115.8751365 E | rice paddy |
| BZ02 | *Grus vipio* | GV | 2020-12-28 | 29.133513 N | 115.8751365 E | rice paddy |
| BZ03 | *Grus vipio* | GV | 2020-12-28 | 29.133513 N | 115.8751365 E | rice paddy |
| BZ04 | *Grus vipio* | GV | 2020-12-28 | 29.133513 N | 115.8751365 E | rice paddy |
| BZ05 | *Grus vipio* | GV | 2020-12-28 | 29.133513 N | 115.8751365 E | rice paddy |
| BZ06 | *Grus vipio* | GV | 2020-12-28 | 29.133513 N | 115.8751365 E | rice paddy |
| BZ07 | *Grus vipio* | GV | 2020-12-28 | 29.133513 N | 115.8751365 E | rice paddy |
| BZ08 | *Grus vipio* | GV | 2020-12-28 | 29.133513 N | 115.8751365 E | rice paddy |
| BZ09 | *Grus vipio* | GV | 2020-12-28 | 29.133513 N | 115.8751365 E | rice paddy |
| BZ11 | *Grus vipio* | GV | 2020-12-28 | 29.133513 N | 115.8751365 E | rice paddy |
| BZ13 | *Grus vipio* | GV | 2020-12-28 | 29.133513 N | 115.8751365 E | rice paddy |
| BZ14 | *Grus vipio* | GV | 2020-12-28 | 29.133513 N | 115.8751365 E | rice paddy |
| BZ15 | *Grus vipio* | GV | 2020-12-28 | 29.133513 N | 115.8751365 E | rice paddy |
| BZ16 | *Grus vipio* | GV | 2020-12-28 | 29.133513 N | 115.8751365 E | rice paddy |
| BZ24 | *Grus vipio* | GV | 2020-12-28 | 29.133513 N | 115.8751365 E | rice paddy |
| HH01 | *Grus grus* | GG | 2020-11-21 | 28.77654 N | 116.303286 E | rice paddy |
| HH02 | *Grus grus* | GG | 2020-11-21 | 28.77654 N | 116.303286 E | rice paddy |
| HH03 | *Grus grus* | GG | 2020-11-21 | 28.77654 N | 116.303286 E | rice paddy |
| HH04 | *Grus grus* | GG | 2020-11-21 | 28.77654 N | 116.303286 E | rice paddy |
| HH05 | *Grus grus* | GG | 2020-11-21 | 28.77654 N | 116.303286 E | rice paddy |
| HH06 | *Grus grus* | GG | 2020-11-21 | 28.77654 N | 116.303286 E | rice paddy |
| HH07 | *Grus grus* | GG | 2020-11-21 | 28.77654 N | 116.303286 E | rice paddy |
| HH08 | *Grus grus* | GG | 2020-11-21 | 28.77654 N | 116.303286 E | rice paddy |
| HH09 | *Grus grus* | GG | 2020-11-21 | 28.77654 N | 116.303286 E | rice paddy |
| HH10 | *Grus grus* | GG | 2020-11-21 | 28.77654 N | 116.303286 E | rice paddy |
| HH11 | *Grus grus* | GG | 2020-11-21 | 28.77654 N | 116.303286 E | rice paddy |
| HH12 | *Grus grus* | GG | 2020-11-21 | 28.77654 N | 116.303286 E | rice paddy |

**Note:** Values <0.01% indicate detected relative abundances below 0.01%; 0.00% indicates that the taxon was not detected.

**Table S2 Taxa annotated as potentially pathogen-associated and the literature sources used for their classification.**

| **Taxonomic rank** | **Taxon** | **LL** | **GM** | **GV** | **GG** | **References** |
| --- | --- | --- | --- | --- | --- | --- |
| *Genus* | *Clostridium* | 9.48% | 2.70% | 6.65% | 3.49% | Mohd Shaufi, M. A., Sieo, C. C., Chong, C. W., Gan, H. M., & Ho, Y. W. (2015). Deciphering chicken gut microbial dynamics based on high-throughput 16S rRNA metagenomics analyses. Gut Pathogens, 7(1), 4. https://doi.org/10.1186/s13099-015-0051-7 |
| *Genus* | *Prevotella* | 1.14% | 2.82% | 0.13% | 2.80% | Thomas, F., Hehemann, J.-H., Rebuffet, E., Czjzek, M., & Michel, G. (2011). Environmental and gut Bacteroidetes: The food connection. Frontiers in Microbiology, 2, 93. https://doi.org/10.3389/fmicb.2011.00093 |
| *Genus* | *Aeromonas* | 0.03% | 0.01% | 0.04% | 5.63% | Rodríguez, I., Novoa, B., & Figueras, A. (2008). Immune response of zebrafish (*Danio rerio*) against a newly isolated bacterial pathogen Aeromonas hydrophila. Fish & Shellfish Immunology, 25(3), 239–249. https://doi.org/10.1016/j.fsi.2008.05.002 |
| *Genus* | *Streptococcus* | 0.38% | 0.98% | 0.14% | 0.38% | Shang, W., Li, S., Zhang, L., Wu, H., & Jiang, Y. (2020). The composition of gut microbiota community structure of Jankowski’s bunting (*Emberiza jankowskii*). Current Microbiology, 77(11), 3731–3737. https://doi.org/10.1007/s00284-020-02048-6 |
| *Genus* | *Mycobacterium* | 0.09% | 1.00% | 0.10% | 0.01% | Skeen, H. R., Cooper, N. W., Hackett, S. J., Bates, J. M., & Marra, P. P. (2021). Repeated sampling of individuals reveals impact of tropical and temperate habitats on microbiota of a migratory bird. Molecular Ecology, 30(22), 5900–5916. https://doi.org/10.1111/mec.16170 |
| *Genus* | *Vibrio* | 0.06% | 0.09% | 0.01% | 0.06% | Kim, H.-J., Ryu, J.-O., Lee, S.-Y., Kim, E.-S., & Kim, H.-Y. (2015). Multiplex PCR for detection of the Vibrio genus and five pathogenic Vibrio species with primer sets designed using comparative genomics. BMC Microbiology, 15(1), 239. https://doi.org/10.1186/s12866-015-0577-3 |
| *Genus* | *Corynebacterium* | 0.02% | 0.02% | 0.06% | 0.11% | Grond, K., Sandercock, B. K., Jumpponen, A., & Zeglin, L. H. (2018). The avian gut microbiota: Community, physiology and function in wild birds. Journal of Avian Biology, 49(11), e01788. https://doi.org/10.1111/jav.01788 |
| *Genus* | *Rhodopseudomonas* | 0.01% | 0.10% | 0.01% | 0.00% | Tang, K.-Y., Wang, Z.-W., Wan, Q.-H., & Fang, S.-G. (2019). Metagenomics reveals seasonal functional adaptation of the gut microbiome to host feeding and fasting in the Chinese alligator. Frontiers in Microbiology, 10, 2409. https://doi.org/10.3389/fmicb.2019.02409 |
| *Genus* | *Proteus* | 0.00% | 0.00% | 0.06% | 0.00% | Younis, K. M., Usup, G., & Ahmad, A. (2016). Secondary metabolites produced by marine Streptomyces as antibiofilm and quorum-sensing inhibitor of uropathogen Proteus mirabilis. Environmental Science and Pollution Research, 23(5), 4756–4767. https://doi.org/10.1007/s11356-015-5687-9 |
| *Genus* | *Fusobacterium* | 0.01% | 0.00% | 0.01% | 0.04% | Laviad-Shitrit, S., Izhaki, M., Lalzar, M., & Halpern, M. (2019). Comparative analysis of intestine microbiota of four wild waterbird species. Frontiers in Microbiology, 10, 1911. https://doi.org/10.3389/fmicb.2019.01911 |
| *Genus* | *Rhodococcus* | 0.00% | 0.05% | 0.00% | 0.00% | von Bargen, K., & Haas, A. (2009). Molecular and infection biology of the horse pathogen Rhodococcus equi. FEMS Microbiology Reviews, 33(5), 870–891. https://doi.org/10.1111/j.1574-6976.2009.00181.x |
| *Genus* | *Pseudomonas* | 0.01% | 0.04% | 0.00% | 0.00% | Levesque, B., Brousseau, P., Bernier, F., & Joly, J. R. (2000). Study of the bacterial content of ring-billed gull droppings in relation to recreational water quality. Water Research, 34(4), 1089–1096. https://doi.org/10.1016/S0043-1354(99)00266-3 |
| *Genus* | *Campylobacter* | 0.00% | 0.00% | 0.00% | 0.01% | Lewis, W. B., Moore, F. R., & Wang, S. (2017). Changes in gut microbiota of migratory passerines during stopover after crossing an ecological barrier. The Auk: Ornithological Advances, 134(1), 137–145. https://doi.org/10.1642/AUK-16-120.1 |
| *Genus* | *Haemophilus* | 0.00% | 0.00% | 0.00% | 0.01% | Murphy, T. F., Faden, H. S., Bakaletz, L. O., & Pelton, S. I. (2009). *Nontypeable Haemophilus* influenzae as a pathogen in children. The Pediatric Infectious Disease Journal, 28(1), 43–48. https://doi.org/10.1097/INF.0b013e318184dba2 |
| *Genus* | *Staphylococcus* | 0.00% | 0.01% | 0.00% | 0.00% | Mushtaq, M., Bukhari, S. M., Ahmad, S., Khattak, A., Chattha, M. B., et al. (2023). Isolation and characterization of bacteria residing in the oral, gut, and fecal samples of different pheasant species. Brazilian Journal of Biology, 83, e249159. https://doi.org/10.1590/1519-6984.249159 |
| *Species* | *Mucispirillum schaedleri* | 0.05% | 0.06% | 0.00% | 0.07% | Herp, S., Durai Raj, A. C., Salvado Silva, M., Woelfel, S., & Stecher, B. (2021). The human symbiont *Mucispirillum schaedleri*: Causality in health and disease. Medical Microbiology and Immunology, 210(4), 173–179. https://doi.org/10.1007/s00430-021-00702-9 |
| *Species* | *Prevotella melaninogenica* | 0.00% | 0.04% | 0.01% | 0.01% | Larsen, J. M. (2017). The immune response to *Prevotella* bacteria in chronic inflammatory disease. Immunology, 151(4), 363–374. https://doi.org/10.1111/imm.12760 |
| *Species* | *Rothia dentocariosa* | 0.00% | 0.01% | 0.00% | 0.00% | Norberg, A. N., Norberg, P. R. B. M., Manhães, F. C., Mangiavacchi, B. M., Ferreira Filho, R. M., Arêas, J. T. C., et al. (2023). Microbiological and epidemiological aspects of endocarditis caused by Rothia spp.: A systematic review. Journal of Advances in Medicine and Medical Research, 35(24), 100–127. https://doi.org/10.9734/jammr/2023/v35i245328 |

Values are mean relative abundances within each host species. Genus-level annotations may include both pathogenic and non-pathogenic members, and detection by 16S rRNA gene sequencing does not confirm pathogenicity, infection, or disease.
